# Editorial: The effect of muscle pathogenesis on avian physiology, animal welfare, and quality of muscle as a food

**DOI:** 10.3389/fphys.2025.1577465

**Published:** 2025-03-10

**Authors:** Sandra G. Velleman, Wei Guo, Gale M. Strasburg

**Affiliations:** ^1^ Department of Animal Sciences, The Ohio State University, Wooster, OH, United States; ^2^ Department of Animal and Dairy Sciences, University of Wisconsin-Madison, Madison, WI, United States; ^3^ Department of Food Science and Human Nutrition, Michigan State University, East Lansing, MI, United States

**Keywords:** metabolomics, muscle physiology, myopathy, spaghetti meat, wooden breast, welfare

## Introduction

The effect of muscle pathogenesis on avian physiology, animal welfare, and quality of muscle as a food Research Topic was aimed at highlighting research focused on the physiological mechanisms of muscle pathogenesis in poultry, which can alter bird survival and the quality of muscle as food. A total of 11 submissions were accepted for publication, covering the areas of wooden breast and spaghetti meat, meat quality and welfare, immune system function and fibroadipogenic progenitors, and the merging of modern omics research with hypothesis-based research.

## Wooden breast and spaghetti meat

Six manuscripts within the Research Topic are devoted to wooden breast and spaghetti meat myopathies. Rimmer and Zumbaugh provided a review of the current status of wooden breast, with attention paid to biochemical pathways involved in the conversion of muscle to meat. Alteration of these pathways may result in the high ultimate pH found in wooden breast meat. They suggested altered mitochondria function may exist in wooden breast-affected muscle. Supporting evidence comes from an original research study by Greene et al., who compared mitochondria isolated from wooden breast muscle with that of hypoxic chicken myoblasts and found Ca^2+^ overload and other features of mitochondrial dysfunction. Their results suggest that aberrant Ca^2+^ signaling associated with chronic local hypoxia may be a primary contributor to the etiology of the wooden breast myopathy. Two original research contributions by Pejšková et al. focused on extracellular matrix remodeling and the expression of transmembrane heparan sulfate proteoglycans, the syndecans. Since syndecans are transmembrane proteoglycans, they form a bridge between the external extracellular matrix and the internal cellular environment. Syndecan-4 plays a significant role in muscle regeneration by regulating the proliferation of muscle satellite cells. They reported decreased syndecan-4 expression and increased shedding of the ectodomain, leading to decreased cell proliferation affecting the regeneration of damaged muscle fibers. Furthermore, expression and oligomerization patterns of syndecans-1 through 3 also changed with wooden breast, affecting key cellular signal transduction pathways like MAPK, AKT, and Wnt.

Che and Hall and Choi et al. contributed an Opinion paper and original research contribution, respectively, comparing spaghetti meat to wooden breast. Both spaghetti meat and wooden breast are two myopathies resulting in economic losses for the poultry industry by affecting the broiler breast meat quality. Spaghetti meat results in the separation of muscle fibers, leading to a spaghetti-type phenotype, whereas wooden breast is characterized by a hard breast muscle. Che and Hall highlighted that the differences in heritability and processing factors require multifaceted approaches, including genetic selection, management practices, and processing methods. Choi et al. utilized a comparative metabolomic analysis to identify similarities and differences between wooden breast and spaghetti meat. In their study, they identified 3,090 metabolites in the chicken breast. In wooden breast, 850 metabolites are affected, whereas in spaghetti meat, 617 metabolites are differential. There were differences in the differential metabolites between the two myopathies, with cellular homeostasis and lipid metabolism both being affected in wooden breast and spaghetti meat. For example, purine metabolism was upregulated, and folic acid was decreased. They suggested that dietary supplementation of appropriate nutrients like folic acid may be a strategy to reduce the incidence or severity of wooden breast and spaghetti meat.

## Meat quality and welfare

Maynard et al. reported on the effects of *in ovo* nicotinamide riboside feeding of high-yield Cobb 700 broilers for growth and meat quality. Nicotinamide riboside is a vitamin B3 analog that may protect against muscle degeneration. In general, nicotinamide ribose dosing increased pectoralis major and minor weights coupled with increased muscle fiber numbers. Despite the increase in weight and muscle fibers, myopathy scores and incidence of wooden breast were minimally affected. Anthney et al. reviewed bacterial chondronecrosis with osteomyelitis lameness and its effects on broiler welfare and meat quality. As a welfare issue, bacterial chondronecrosis leads to lameness which is considered painful to the bird but also results in the loss of product from culls and condemnations. Frequently, birds with bacterial chondronecrosis will be targets of aggression from more dominant littermates. Meat quality is a concern due to possible microbial contamination, especially in birds whose internal lesions have not been detected during carcass processing.

## Immune system and fibroadipogenic progenitors

Song et al. conducted a comparative transcriptomic analysis of organs affected by Marek’s disease virus and found cytokine–cytokine receptor interactions and cellular development were affected by Marek’s disease. Lesions in wooden breast and white striping are characterized by the replacement of healthy tissue with fibrous and fatty tissue deposits. Fibroadipogenic progenitors are interstitial muscle mesenchymal stem cells and differentiate into both adipocytes and fibroblasts. To date, fibroadipogenic progenitors have been identified in myopathies in mice and humans. Jung et al. reviewed evidence that the fibroadipogenic population of cells may be a novel cell type and new target to direct research in chickens to reduce the incidence of breast muscle myopathies.

## Merging of modern omics research with hypothesis-based research

Guo et al. provided an Opinion paper exploring how we have shifted to new omics technologies scientifically that have moved us to hypothesis-generating research. Traditionally, science made incremental advances through hypothesis-based research. The authors discuss how a balance and integration of both strategies is needed to translate new molecular discoveries into biological mechanistic insights and applications.

